# Developmental maturation of millimeter-scale functional networks across brain areas

**DOI:** 10.1093/cercor/bhaf007

**Published:** 2025-01-25

**Authors:** Nathaniel J Powell, Bettina Hein, Deyue Kong, Jonas Elpelt, Haleigh N Mulholland, Ryan A Holland, Matthias Kaschube, Gordon B Smith

**Affiliations:** Optical Imaging and Brain Sciences Medical Discovery Team, Department of Neuroscience, University of Minnesota, 2021 6th St. SE, Minneapolis, MN 55455, United States; Center for Theoretical Neuroscience, Columbia University, 3227 Broadway, New York, NY 10027, United States; Frankfurt Institute for Advanced Studies, Ruth-Moufang-Str. 1, 60438 Frankfurt am Main, Germany; Department of Computer Science and Mathematics, Goethe University Frankfurt, Robert-Mayer-Str. 10, 60054 Frankfurt am Main, Germany; International Max Planck Research School for Neural Circuits, Max-von-Laue-Str. 4, 60438 Frankfurt am Main, Germany; Frankfurt Institute for Advanced Studies, Ruth-Moufang-Str. 1, 60438 Frankfurt am Main, Germany; Department of Computer Science and Mathematics, Goethe University Frankfurt, Robert-Mayer-Str. 10, 60054 Frankfurt am Main, Germany; Optical Imaging and Brain Sciences Medical Discovery Team, Department of Neuroscience, University of Minnesota, 2021 6th St. SE, Minneapolis, MN 55455, United States; Optical Imaging and Brain Sciences Medical Discovery Team, Department of Neuroscience, University of Minnesota, 2021 6th St. SE, Minneapolis, MN 55455, United States; Frankfurt Institute for Advanced Studies, Ruth-Moufang-Str. 1, 60438 Frankfurt am Main, Germany; Department of Computer Science and Mathematics, Goethe University Frankfurt, Robert-Mayer-Str. 10, 60054 Frankfurt am Main, Germany; Optical Imaging and Brain Sciences Medical Discovery Team, Department of Neuroscience, University of Minnesota, 2021 6th St. SE, Minneapolis, MN 55455, United States

**Keywords:** calcium imaging, cortex, development, ferret, spontaneous activity

## Abstract

Processing sensory information, generating perceptions, and shaping behavior engages neural networks in brain areas with highly varied representations, ranging from unimodal sensory cortices to higher-order association areas. In early development, these areas share a common distributed and modular functional organization, but it is not known whether this undergoes a common developmental trajectory, or whether such organization persists only in some brain areas. Here, we examine the development of network organization across diverse cortical regions in ferrets using in vivo wide field calcium imaging of spontaneous activity. In both primary sensory (visual, auditory, and somatosensory) and higher order association (prefrontal and posterior parietal) areas, spontaneous activity remained significantly modular with pronounced millimeter-scale correlations over a 3-wk period spanning eye opening and the transition to externally-driven sensory activity. Over this period, cortical areas exhibited a roughly similar set of developmental changes, along with area-specific differences. Modularity and long-range correlation strength generally decreased with age, along with increases in the dimensionality of activity, although these effects were not uniform across all brain areas. These results indicate an interplay of area-specific factors with a conserved developmental program that maintains modular functional networks, suggesting modular organization may be involved in functional representations in diverse brain areas.

## Introduction

In the mature cortex, neurons selective for features of the internal or external environment participate in highly interconnected neural networks that provide the basis for perception and behavior. Across a range of cortical areas, the information processed by these networks varies greatly, from primarily unimodal sensory representations in areas such as A1, V1, and S1, to more complex and higher order representations in areas such as posterior parietal cortex (PPC) and prefrontal cortex (PFC). The developmental origin of this functional diversity is a central question in cortical development, as is the relative balance of common organizational features versus early area-specific functions. Current evidence suggests that the specialization of cortical areas is thought to begin early in development, with genetic cues defining broad area identity, which is later refined through area-specific inputs in an activity dependent manner (Sur and Rubenstein 2005; Cadwell et al. 2019), although the degree to which this cortical maturation occurs in concert across diverse brain areas remains unclear.

Much of what is known about the developmental maturation of functional properties at the network level comes from studies of primary sensory cortices. Here, the properties of the mature network emerge over the course of development through considerable refinement at both the neuron and network level. For example, in ferret V1, from the time neurons can be driven by visual stimuli they become increasingly selective to stimulus features such as edge orientation (Chapman and Stryker 1993) and direction of motion (Li et al. 2006). In carnivores such as the ferret and cat, as well as in primates, these representations are organized into a modular (or “columnar”) structure, with nearby neurons exhibiting similar selectivity for a range of visual properties which vary across the cortex, giving rise to distributed patches of co-active neurons (Hubel and Wiesel 1968; Blasdel and Salama 1986; Weliky et al. 1996; Issa et al. 2000; Kara and Boyd 2009; Smith et al. 2015a). Although the spacing of modular activity can vary across both visual properties (Nauhaus et al. 2016), areas (Kaschube et al. 2009) and species (Kaschube et al. 2010), the wavelength is typically on the order of 0.5 to 2 mm, reflecting the presence of millimeter-scale functional networks.

Several studies in the ferret have uncovered key features of the developmental emergence of this functional organization, with orientation preference maps emerging around the time of eye opening (Chapman et al. 1996). However, well before the onset of these sensory-evoked responses, functional neural networks exist in the visual cortex that are readily apparent through correlations in spontaneous activity (Chiu and Weliky 2001). Activity in these early networks shows a highly dense and pronounced modular spatial structure, with nearby neurons showing highly coherent activity patterns that are organized into distributed functional networks comprised of multiple modules correlated across millimeters (Smith et al. 2018). Notably, these early correlated networks appear to be a precursor for the modular representation of orientation (Smith et al. 2018; Trägenap et al. 2023).

We recently found that in the ferret during early development, such distributed and modular functional networks are not unique to V1, but are a common feature that is shared across diverse cortical areas. Both the primary sensory areas A1, S1, and V1, as well as the higher-order association areas PFC and PPC exhibit spontaneous activity that is strongly correlated among local populations, forming functional modules which themselves form patchy millimeter-scale distributed networks with wavelengths near 1 mm and correlations extending over several millimeters. (Powell et al. 2024). To date, these modular functional networks outside V1 have only been examined during early development, well before the onset of mature sensory-evoked responses and the key developmental transition to externally-driven sensory-evoked activity. Thus, it remains unclear whether the continued presence of modular functional organization seen in V1 is indicative of a visual-specific process, or rather is a reflection of a more global and universal developmental program shared across the cortex.

To address this question, we used ongoing spontaneous activity recorded under light anesthesia to assess functional network organization across the cortex at two key periods in development: first, around the time of eye-opening and ear canal opening in the ferret (P27–32), which marks a major developmental milestone and a transition in the nature of incoming sensory information; and secondly 1 to 2 wk later (P39–43), when response properties in V1 have been shown to approach mature levels (Li et al. 2006; Smith et al. 2015b). Examining spontaneous activity allows us to measure and compare intrinsic network organization across areas, without the need to design optimal stimuli for both sensory and non-sensory areas. Comparing this data to that which we previously obtained from P21–24 (Powell et al. 2024), we find that the developmental maturation of network properties reflects an interplay between area-specific processes and a common developmental program. Across all developmental ages examined, we find that spontaneous activity remained significantly modular in both sensory and non-sensory cortical areas. In general, across areas we observed a similar weakening of modular organization, while spontaneous activity continued to show distributed and modular correlations across millimeters. Notably, overall the strength of millimeter-scale correlations decreased and activity became higher dimensional with age, with roughly similar changes in PFC, PPC, S1 and A1. However, V1 showed clear area-specific differences, with millimeter-scale correlations remaining high with age. Together, these results demonstrate that the modular functional organization that serves as a common developmental origin for highly diverse cortical areas (Powell et al. 2024) remains a common feature of functional activity across areas even as they undergo developmental trajectories reflecting both shared and area-specific elements.

## Materials and methods

### Data collection

#### Animals

All experimental procedures were approved by the University of Minnesota Institutional Animal Care and Use Committee (protocol ID 2306-41147A) and were performed in accordance with guidelines from the US National Institutes of Health. We obtained 27 male and female ferret kits from Marshall Farms and housed them with jills on a 16-h light/8-h dark cycle. No statistical methods were used to predetermine sample sizes, but our sample sizes are similar to those reported in previous publications (e.g. Chapman et al. 1996; Smith et al. 2015b; Wilson et al. 2017; Smith et al. 2018; Stacy et al. 2023).

#### Viral injection

Viral injections were performed largely as described (Smith and Fitzpatrick 2016; Powell et al. 2024). Briefly, we expressed GCaMP6s (Chen et al. 2013) under the synapsin promoter by microinjecting AAV1.hSyn.GCaMP6s.WPRE.SV40 (Addgene) into layer 2/3 of targeted cortical areas at P13–32, 7 to 21 d before imaging. In ferrets injected at prior to p26, 7 d of expression has been shown to be sufficient to achieve robust expression of GCaMP6s (Smith et al. 2015b; Smith et al. 2018). Anesthesia was induced with isoflurane (4–5%) and maintained with isoflurane (1–1.5%). Glycopyrrolate (0.01 mg/kg) or Atropine (0.02 mg/kg) and bupivacaine/lidocaine (1:1 mixture) were administered, and animal temperature was maintained at approximately 37°C with a water pump heat therapy pad (Adroit Medical HTP-1500, Parkland Scientific). Animals were mechanically ventilated and both heart rate and end-tidal CO_2_ were monitored throughout the surgery (Digicare LifeWindow). Using aseptic surgical technique, skin and muscle overlying target areas were retracted, and a small burr hole (~1 mm) was made with a handheld drill (Fordom Electric Co.). Approximately 1 μL of virus contained in a pulled-glass pipette was pressure injected into the cortex at two or three depths (~200 to 400 μm below the surface) over 20 min using a Nanoject-III (World Precision Instruments). The craniotomy was then sealed and the skin sutured closed. In both these experiments and as reported previously (Smith et al. 2018), this procedure typically resulted in robust expression of GCaMP over a region approximately 3–4 mm in diameter, with fairly uniform expression over this region and a gradual fall-off in expression at greater distances.

Coordinates for targeted injections were as in (Powell et al. 2024): (coordinates relative to Bregma): PFC: ~ 1–2 mm lateral, ~ 7–8 mm anterior; PPC: ~ 1–2 mm lateral, ~ 4 mm posterior; S1: ~ 2–3 mm lateral, ~ 1 mm anterior; A1: ~ 7–9 mm lateral, ~ 3 mm posterior. V1 was targeted relative to Lamda: ~ 6–8 mm lateral, ~ 1–2 mm anterior. We injected virus into and imaged 1 to 3 areas per animal. In most experiments, multiple areas were labeled and imaged in a single animal (mean 1.9 ± 0.2 areas per animal).

#### Cranial window surgery

Cranial window implants were performed largely as described (Smith and Fitzpatrick 2016; Powell et al. 2024). On the day of imaging, ferrets aged P27–43 were anesthetized with isoflurane (4% to 5% for induction, 1% to 2% maintenance) and atropine (0.2 mg/kg) or glycopyrrolate (0.01 mg/kg) was administered. Animals were placed on a feedback-controlled heating pad to maintain an internal temperature of 37°C. Animals were intubated and ventilated. Isoflurane was delivered between 1% and 2% throughout the surgical procedure to maintain a surgical plane of anesthesia. An intraperitoneal or intravenous catheter was placed to deliver fluids. EKG, end tidal CO_2_, and internal temperature were continuously monitored during the procedure and subsequent imaging session. The scalp was retracted and a custom titanium head mount adhered to the skull using C&B Metabond (Parkell). A craniotomy was performed by manually drilling a 6 to 7 diameter mm circle with a small drill bit (0.5 mm carbide burr, Komet USA) over areas of viral expression and the dura was retracted to reveal the cortex. Cover glass (round, #1.5 thickness, Electron Microscopy Sciences) adhered to the bottom of a custom titanium or 3D printed plastic insert was placed onto the brain to gently compress the underlying cortex and dampen biological motion during imaging. Surgical procedures typically lasted 1 to 3 h. Upon completion of the surgical procedure, isoflurane was gradually reduced (0.6 to 1.0%) and then vecuronium bromide (2 mg/kg/h) was delivered to reduce motion and prevent spontaneous respiration.

#### Wide field epifluorescence imaging

Beginning approximately 1 to 2 h after the end of the surgical procedure, spontaneous activity was recorded in a quiet darkened room for 10 to 40 min per area. In these experiments, the necessity of performing recordings in young animals at specific developmental ages precluded the lengthy periods of habituation required for awake head-fixed imaging. For this reason, and also to maintain consistency with prior work which utilized similar experimental conditions (Powell et al. 2024), imaging was performed under light isoflurane anesthesia (0.6% to 1%). In prior experiments in V1, these conditions were shown to not alter the spatial structure of modular functional activity or its long-range correlations (Smith et al. 2018). Wide field epifluorescence imaging was performed (except for 1 animal, see below) with 4x objective (Olympus) using a Zyla 5.5 sCMOS camera (Andor) mounted to the camera port of a commercial microscope assembly (Neurolabware) and controlled by MicroManager Software (Edelstein et al. 2014). One animal was imaged with a custom built tandem-lens macroscope (Ratzlaff and Grinvald 1991), using the same Zyla sCMOS camera and MicroManager Software. Images were acquired at 15 Hz with 4 × 4 binning to yield 640 × 540 pixels, spanning a field of view of ~ 3.5 × 3 mm.

#### Histology

**Fig. 1:**
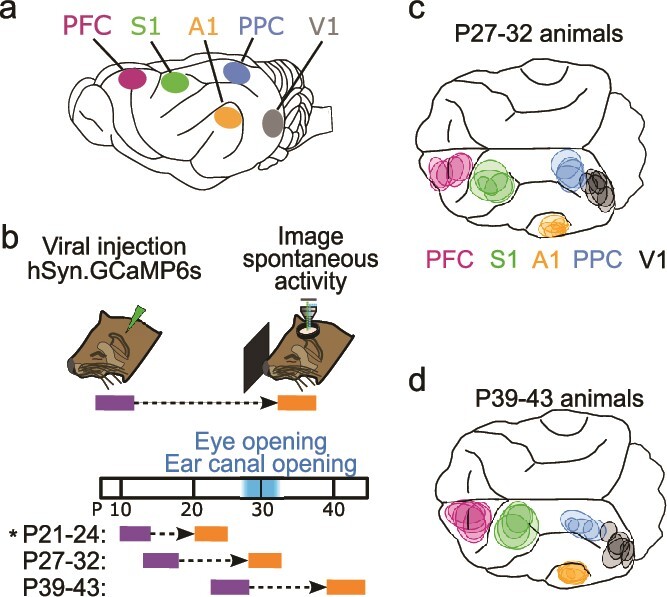
Imaging distinct cortical regions in ferrets at different developmental ages. a) Target cortical locations. S1—somatosensory cortex, A1—auditory cortex, V1—visual cortex. b) Experimental timeline. Animals were injected with AAV expressing GCaMP6s 10 to 14 d prior to imaging. Imaging was performed at P27–32 or P39–43. P21–24 data from Powell et al. 2024 (indicated by ^*^) is presented for comparison with other ages. c) Imaged locations for P27–32 animals reconstructed from histology, colored based on assigned cortical area. d) Same as c for P39–43 animals.

Following imaging, animals were euthanized with 5% Isoflurane and pentobarbital. Animals were perfused with heparinized saline solution followed by 4% paraformaldehyde, then the brains were removed and kept for histology. Viral expression of GCaMP was documented in the intact brain with appropriate excitation and emission filters. Images of expression were aligned to a common coordinate system using prominent brain features (sulci, fissures, and external edges of brain). Areas of expression from each brain were outlined manually in Matlab, and are shown in Fig. 1.

### Analysis methods

#### Wide field data pre-processing

Wide field data pre-processing, event extraction and calculation of spontaneous correlations was performed largely as described (Smith et al. 2018; Powell et al. 2024) for all imaged areas. Briefly, brain motion during imaging was corrected by performing rigid image registration and a region of interest (ROI) was manually drawn around the cortical area with high and robust spontaneous activity. ROIs were also drawn to remove any artifacts or debris in the visible field of view (FOV). The baseline fluorescence (F_0_) for each pixel was obtained by applying a median filter to the raw fluorescence trace with a window between 10 and 64 s. Filter width was chosen for each imaging session individually, such that the baseline followed faithfully the slow trend of the fluorescence activity. The baseline corrected activity was calculated as


(1) \begin{equation*} \left(F-{F}_0\right)/{F}_0=\Delta \mathrm{F}/{\mathrm{F}}_0 \end{equation*}

#### Event detection

To detect spontaneously active events, we utilized the same approach as (Smith et al. 2018; Powell et al. 2024), which was designed to identify instances of large spontaneous activity, termed “events.” First, we fit a standard Gaussian to the activity of each pixel over time. In order to exclude large positive values (putative events) from the determination of mean and standard deviation, we binned the data and then truncated the data used for the Gaussian fit at 1.5 times the highest frequency bin. We then determined active pixels on each frame using a pixel-wise threshold set to 3 standard deviations above each pixel’s mean value, thereby detecting high activity over a fluctuating baseline (Supplemental Fig. 1). In the oldest age group (P39–43) this approach failed to accurately detect many events due to a broadening of the distribution of pixel-wise ΔF/F values, leading to a potential bias toward only the largest events. To avoid this, we instead used a pixel-wise threshold set at the 80^th^ percentile of each pixel’s ∆F/F trace in P39–43 animals (Supplemental Fig. 2). Importantly, regardless of the pixel threshold chosen, the detected events were highly modular in all areas at all ages, and the choice of threshold did not qualitatively change the results for modularity or wavelength (described below) (Supplemental Fig. 3). Following these pixel-wise thresholds, we next applied two spatial criteria to define active frames. First, active pixels not part of a contiguous active region of at least 0.01mm^2^ were considered “inactive” for the purpose of event detection in order to minimize the effects of imaging noise which varies independently across pixels. Active frames were taken as frames with a spatially extended pattern of activity (>40% of pixels within ROI were active).

Consecutive active frames were combined into a single event starting with the first high activity frame and then either ending with the last high activity frame or, if present, an activity frame defining a local minimum in the fluorescence activity. In order to assess the spatial pattern of an event, we extracted the maximally active frame for each event (the “event frame”), which was defined as the frame with the highest activity averaged across the ROI. This approach of both pixel-wise and spatial thresholds robustly identified large spontaneous events across different brain areas and ages.

#### Calculation of correlation patterns

To assess the spatial correlation structure of spontaneous or evoked activity (Smith et al. 2018; Mulholland et al. 2021; Powell et al. 2024), we applied a Gaussian spatial band-pass filter (with SD of Gaussian filter kernel *s*_high_ = 195 μm, s_low_ = 26-41 μm) to each event frame and down-sampled it to 160 x 135 pixels. The resulting patterns, named *A_i_* in the following, where i = 1,…,N, and N being the number of patterns detected, were used to compute the spontaneous correlation patterns as the pairwise Pearson’s correlation between all locations ***x*** within the ROI and the seed point ***s***


(2) \begin{equation*} C\left(\boldsymbol{x},\boldsymbol{s}\right)=\frac{1}{N}\frac{\sum_{i=1}^N\left({A}_i\left(\boldsymbol{s}\right)-<{A}_i\left(\boldsymbol{s}\right)>\right)\left({A}_i\left(\boldsymbol{x}\right)-<{A}_i\left(\boldsymbol{x}\right)>\right)}{\sigma_x{\sigma}_s} \end{equation*}

Here the brackets < > denote the average over all *N* patterns and *σ*_***x***_ denotes the standard deviation of *A* over all *N* patterns at location ***x***.

#### Shuffled control ensemble and surrogate correlation patterns

To evaluate the statistical significance of quantities characterizing the correlation patterns observed during spontaneous activity, we compared the real ensemble of spontaneous activity patterns from a given experiment with a control ensemble, obtained by eliminating most of the spatial relationships between the patterns (Smith et al. 2018). To this end, all activity patterns were randomly rotated (rotation angle drawn from a uniform distribution between 0° and 360° with a step size of 10°) and reflected (with probability 0.5, independently at the x- and y-axis at the center of the ROI), resulting in an equally large control ensemble with similar statistical properties, but little systematic interrelation between patterns. Surrogate correlation patterns were then computed from these ensembles as described above.

#### Dimensionality of spontaneous activity

We estimated the cross-validated dimensionality *d*_eff_ of the subspace spanned by spontaneous activity patterns (see (Stringer et al. 2019)). First, we randomly divided the activity patterns into two non-overlapping subsets X_1_ and X_2_, and then performed principal component analysis (PCA) on X_1_ to find the axis representing the principal components (PCs). Next, we projected X_2_ onto these PCs to estimate the variance λ_i_ explained by each PC. Lastly, dimensionality was calculated as (Abbott et al. 2011):


(3) \begin{equation*} {d}_{eff}=\frac{{\left({\sum}_{i=1}^N{\lambda}_i\right)}^2}{\left({\sum}_{i=1}^N{\lambda_i}^2\right)} \end{equation*}

The dimensionality is computed as an average over 10 subsets of 100 randomly chosen events, considering a circular region with area size A = 0.5 Lambda^2 (where Lambda is the median wavelength (computed as described above) over all spontaneous events).

#### Spatial range of correlations

To assess the strength of spontaneous correlations over distance, we computed the variance of pixel-wise correlation values located at a given distance from a seed point (Dahmen et al. 2022; Powell et al. 2024). Closer to the seed point, correlations will have both strongly positive and strongly negative values, leading to a high variance. In contrast, further away from the seed point correlations will be closer to zero and exhibit reduced variance. To assess long-range correlation strength, we computed the variance within a ring from 1.8 to 2.2 mm from seed point. To control for the finite number of events in each experiment, we fixed the number of events to 100. We also computed the variance for surrogate correlation patterns (see above) generated for each experiment using a matched number of patterns to take into account other irregularities (e.g. the shape of the ROI, blood vessel artifacts etc.), thereby allowing comparisons across different experiments from different regions and ages. The ROI for two A1 FOVs were too small to compute a surrogate dataset at 2 mm, and were excluded from this analysis.

#### Modularity and wavelength estimation

To estimate the wavelength of individual calcium events we employed the approach described previously (Powell et al. 2024). Briefly, we calculated the 1-D radial average of the spatial autocorrelation of the band-pass-filtered activity pattern of the event frame, taking the location of the first minimum as one-half the wavelength. The modularity of each event (Powell et al. 2024) was calculated as the absolute difference in amplitude between the first minimum and the subsequent maximum.

To determine if the modularity observed during spontaneous events was statistically significant, we compared it to a distribution of modularity values for inactive frames as in (Powell et al. 2024). Control frames were drawn from the bottom 10% of frames that were not part of an identified spontaneous event (see above) based on mean activity within the ROI. For each experiment, we obtained 100 sets of control frames containing an event-matched number of frames and calculated the median modularity across these frames, generating a distribution of 100 control median modularity values. This was then compared to the median modularity across spontaneous events to obtain a p-value.

#### Module amplitude

The module amplitude of a wide field event (Powell et al. 2024) was taken as the amplitude (in ∆F/F, prior to spatial filtering) of the module peaks divided by the background activity. Peaks were detected using the FastPeakFind.m function in Matlab (Natan 2021), and background activity was taken as the median amplitude of activity in locations one-half wavelength (averaged across all events) from the peak. The module amplitude of an event was taken as the average amplitude across all peaks in the event.

### Statistical analysis

Figures show all data points for each analysis. Parametric summary statistics (mean and standard deviation) are shown in figures and provided for all groups in Supplemental Tables 1, 3, 6, 9, and 11. Nonparametric statistical analyses were used throughout the study for comparisons across groups. All tests were two-sided. Area and age effects and their interactions were tested using the Aligned Rank Transform followed by an analysis of variance (ANOVA) on aligned ranks (ART; Fawcett and Salter 1984; Salter and Fawcett 1985; Wobbrock et al. 2011). Post-hoc tests were performed on transformed data (Elkin et al. 2021), with p values reported using Holm’s correction for multiple comparisons. ART and post-hoc tests were performed in R using the ARTool package (v0.11.0) (Kay et al. 2021). In most cases, p-values are provided within the manuscript text, however in cases in which p-values for multiple comparisons are omitted from the main text to improve readability, all p-values are available in Supplemental Tables 2, 4, 5, 7, 8, 10, and 12.

Data analysis was performed in Matlab (Mathworks), R, and Python.

### Data, materials, and software availability

All data and code used in this study are available upon request from the corresponding author.

## Results

### Distributed and modular spontaneous activity patterns in diverse cortical areas 10–14 d beyond eye-opening

**Fig. 2:**
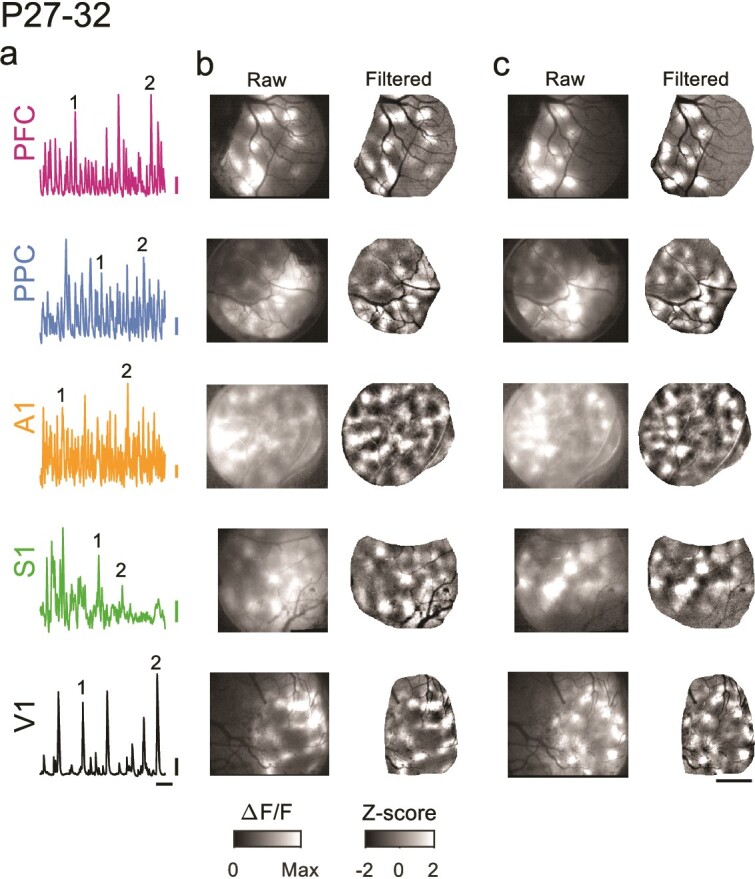
Spontaneous events exhibit widespread and distributed modular activity in diverse brain areas at P27–32. a) Example time course of spontaneous activity in different cortical areas. Numbers indicate events shown in b and c. b) Spontaneous events show modular patterns of activation in PFC, PPC, A1, S1, and V1 at P27–32. Left: raw event pattern showing clear modular patterns of activity in all areas at time (1) in a. Right: same event after applying a high pass spatial filter. c) Second representative event from same experiments as b, at time (2) in a. Scale bars a: 0.1 ∆F/F, 20 s; b and c: 1 mm.

We first sought to examine animals aged P27–32, which in the ferret is around the key developmental milestones of eye- and ear canal-opening (Fig. 1a to c). To assess the state of functional networks at this point in development we expressed the calcium sensor GCaMP6s and examined ongoing spontaneous activity through wide field calcium imaging in five different cortical areas. In both primary sensory (V1, A1, and S1) and association areas (PFC and PPC), we observed frequent spontaneous events that exhibited clear coordination in activity among local populations of neurons (Fig. 2a and b). In all cortical areas, these spontaneous events exhibited clearly modular activity, with distinct patches of activity that were distributed across the cortical surface. Notably this modular structure was evident in unprocessed images without spatial filtering (Fig. 2b and c, left).

**Fig. 3:**
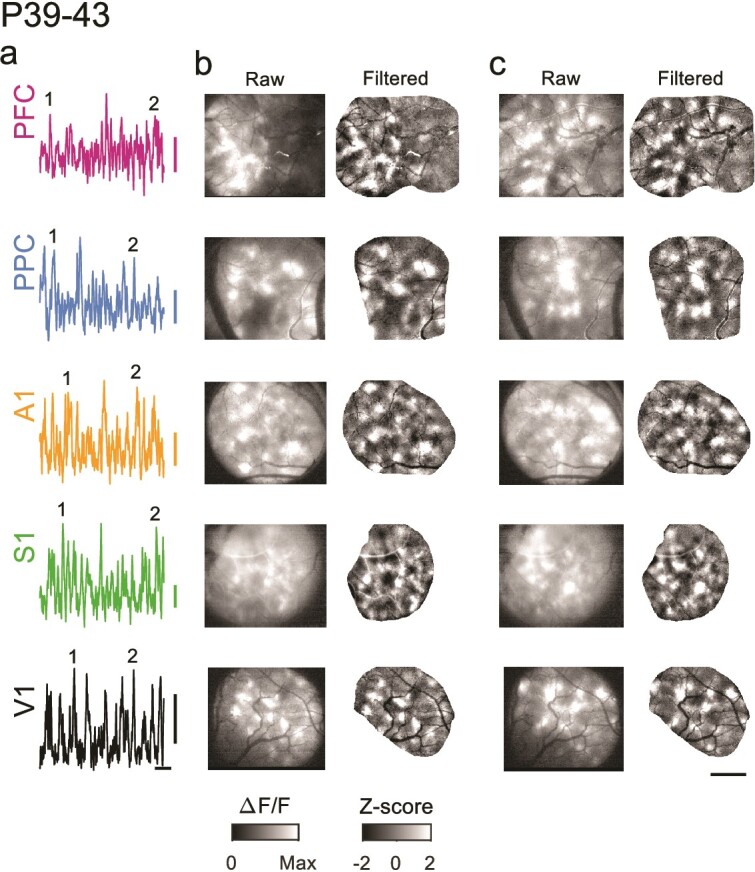
Spontaneous events continue to exhibit widespread and distributed modular activity at P39–43. a) Example time course of spontaneous activity in different cortical areas. Numbers indicate events shown in b and c. b) Spontaneous events show modular patterns of activation in all areas at P39–43. Left: raw event pattern showing clear modular patterns of activity in all areas at time (1) in a. Right: same event after applying a high pass spatial filter. c) Second representative event from same experiments as b, at time (2) in a. Scale bars a: 0.1 ∆F/F, 20 s; b and c: 1 mm.

We next sought to determine whether similarly locally coordinated activity also existed across different cortical areas in animals 1 to 2 wk past eye opening and ear canal opening (P39–43, Fig. 1b and d). Strikingly, in these animals we observed that activity in spontaneous events continued to exhibit clear and pronounced modular structure, with patchy activity distributed across millimeters of cortex (Fig. 3). As in younger animals, the modular structure in spontaneous events was evident in both spatially filtered images (Fig. 3b and c, right), as well as unprocessed images (Fig. 3b and c, left). In general, the overall structure of spontaneous activity patterns was highly similar across areas and ages, and was also similar in appearance to the activity patterns seen previously in animals aged P21–24, 7 to 10 d before eye opening (Powell et al. 2024).

### Quantitative similarity in developmental trajectory of modular spontaneous activity

**Fig. 4:**
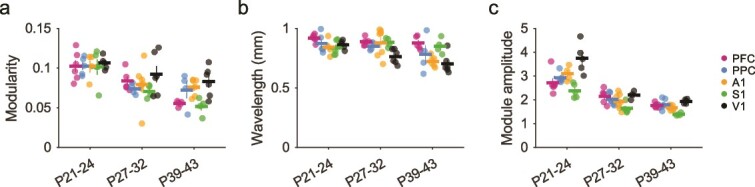
Developmental maturation of modular spontaneous activity across cortical areas. a) Modularity of spontaneous events declines with age, but remains significant in all cases versus controls. b) The wavelength of active modules shows a slight but significant decline with age across areas. c) The amplitude of active modules declines in a similar manner with age in all cortical areas examined. P21–24 data in a to c replotted for comparison from (Powell et al. 2024). In all plots, circles show individual animals, horizontal lines and error bars show mean ± sem.

We next sought to directly compare the strength of modular activity in different brain areas over development. To achieve this, we calculated the modularity of each spontaneous event by first computing the spatial autocorrelation and then measuring the relative amplitude of the first trough and subsequent peak, providing a measure of the regularity in spacing of active domains (Powell et al. 2024). Comparing this data to spontaneous activity at P21–24 (Powell et al. 2024), the strength of this modularity showed a significant decline with age across areas, but did not show a significant interaction of age and area [Fig. 4a, ART, main effect of age F(2) = 29.46, *P* = 1.11 × 10^−9^, main effect of area F(4) = 1.82, *P* = 0.14, interaction F(8) = 1.25, *P* = 0.29]. PFC and S1 showed significant declines in modularity from P21–24 to P39–43 (post-hoc tests with Holm’s correction; PFC: t(61) = 5.04, *P* = 0.0005; S1: t(61) = 4.43, *P* = 0.004, Supplemental Tables 1 and 2). Notably however, events in all cortical areas at both P27–32 and P39–43 remained significantly modular when compared to controls (26 of 26 vs control at P27–32; 26 of 26 significant at P39–43; with *P* < 0.05 in all cases, bootstrap test).

We next measured the spatial wavelength of active domains, again using the spatial autocorrelation of spontaneous events. Overall, the wavelength of activity decreased slightly but significantly with age [Fig. 4b, ART: main effect of age F(2) = 11.23, *P* = 7.04 × 10^−5^], indicating a general progression to higher spatial frequency activity. Our results also show a significant difference in wavelength between cortical areas [ART: main effect of area F(4) = 4.52, *P* = 4.52 × 10^−5^], but did not show a significant interaction of age and area [ART: F(8) = 2.03, *P* = 0.057]. Cross-area post-hoc tests showed a significant difference in wavelength between V1 and both PFC and S1, as well as between PFC and A1 (post-hoc tests with Holm’s correction; V1 vs PFC: t(61) = 5.35, *P* = 7.02 × 10^−6^; V1 vs S1: t(61) = 3.318, *P* = 0.014; PFC vs A1: t(61) = 3.313, *P* = 0.014, Supplemental Tables 3 to 5).

The modularity of events derived from spatial autocorrelations primarily reflects the degree of regularity in the spacing of active domains and is less sensitive to how strongly modules are active relative to surrounding cortex. We therefore computed the “module amplitude” to assess the selectivity of modular activation (see Methods), finding a significant decline with age over all areas [Fig. 4c, ART: main effect of age: F(2) = 82.78, *P* = 2.35^−18^], as well as significant differences across areas, and a significant interaction of age and area [ART: main effect of area: F(4) = 13.28, *P* = 6.64 × 10^−8^; interaction: F(8) = 2.50, *P* = 0.020]. Cross-area post-hoc tests revealed significant differences between module amplitude in V1 versus all other areas, and between S1 and both A1 and PFC (post-hoc tests with Holm’s correction *P* < 0.01 in all cases, see Supplemental Tables 6 and 7). Within area, all regions showed significant declines in module amplitude from P21–24 to P39–43 (post-hoc tests with Holm’s correction *P* < 0.01 in all cases, Supplemental Table 8).

In summary, these results show that the cortical networks in both sensory and association regions continue to exhibit large-scale patterns of distributed modular spontaneous activity well beyond the onset of sensory-evoked responses. Over this period, modular activity showed a general decline in strength and a reduction in wavelength, although the magnitude of these changes was not identical over areas.

### Modular activity shows millimeter-scale correlations in all areas through P39–43

During early development, spontaneous activity not only exhibits modular patterns of activity, but also millimeter-scale correlations (Smith et al. 2018; Powell et al. 2024). The patterns of spontaneous activity at these ages comprise a low dimensional subset of all theoretically possible activity patterns, consistent with certain sets of modules tending to be co-active across events, giving rise to the millimeter scale correlations in activity that are observed in PFC, PPC, A1 and S1 at P21–24 (Powell et al. 2024). We next sought to determine if the observed weakening of modular activity (Fig. 4a and c) is accompanied by a corresponding diversification in millimeter-scale networks, which could result from activity that is locally modular but undergoes a developmental loss in larger-scale organization. Alternatively, large-scale correlated networks could remain present in diverse cortical areas, consistent with multiple studies that have found millimeter-scale modular networks as a prominent feature in V1 in the mature brain (Kenet et al. 2003; O’Hashi et al. 2018; Smith et al. 2018). In order to determine if the large-scale network structure that is common in the early cortex (Powell et al. 2024) follows distinct developmental trajectories in V1 versus other cortical areas, we computed correlations across the spontaneous events observed in wide field imaging.

**Fig. 5:**
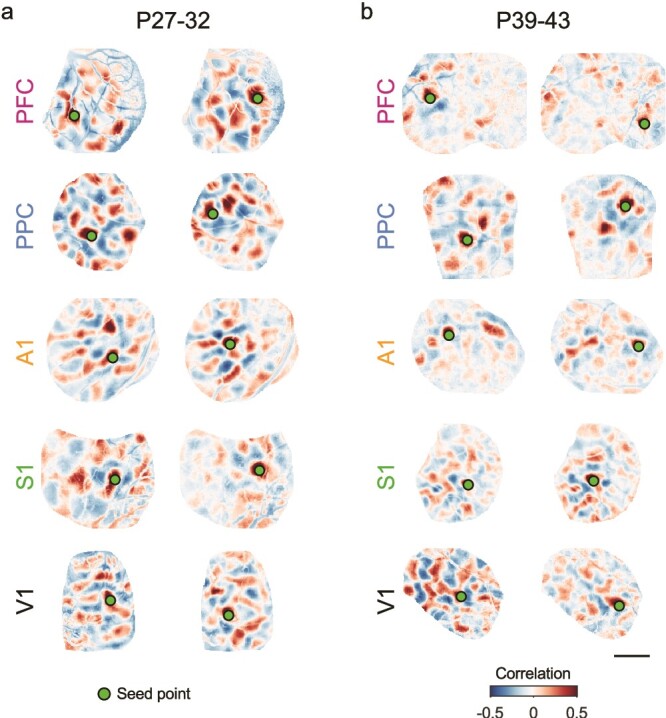
Millimeter-scale modular correlations across diverse brain areas after eye-opening. a) Correlations across spontaneous events for representative experiments at P27–32. Pixel-wise correlations are shown relative to two different seed points for each area. Note that the spatial patterns of correlations vary between seed points, reflecting multiple distinct correlated networks. b) Modular correlations are still present at P39–43 in all cortical areas examined. Scale bar: 1 mm.

We found that in all cortical areas examined, at P27–32, spontaneous activity continued to show long-range correlations, with strongly correlated modules frequently appearing separated by millimeters of cortical area (Fig. 5a). Notably, multiple functional networks were reflected in the structure of these correlations, as evidenced by the diversity in the spatial pattern of correlations for different seed points (Fig. 5a, left vs right). When we examined correlations at P39–43, we observed that the overall structure remained roughly similar in all cortical areas, with positively correlated modules separated by several millimeters. As in younger ages, different cortical locations participated in differently correlated networks (Fig. 5b, left vs right). However, the strength of these correlations appeared to decrease with age in all areas other than V1, with correlation patterns showing generally weaker and less distinct structure than at earlier ages. This progression was qualitatively similar across areas outside of V1, suggesting a similar developmental trajectory across these regions.

### Developmental maturation in millimeter-scale correlated networks across brain areas

We next sought to quantify the strength of these long-range correlations and their developmental progression across areas. Given that the absolute amplitude of correlations can be influenced by the finite number of spontaneous events we are able to observe in each experiment, making direct comparisons of correlation amplitude across areas and ages is challenging. To address this, we employed a measure of correlation strength that allows for the correction of spurious correlations that result from finite event numbers. Thus, correlation strength was determined by computing the variance in pixel-wise correlations (Dahmen et al. 2022; Powell et al. 2024) at a distance of 2 mm (bin size 1.8 to 2.2 mm) from the seed point. Strong correlations will exhibit high variance (as they include both strongly positive and strongly negative peaks), whereas weak correlations will show low variance. Importantly, this approach allows for subtracting the contribution of artifacts in correlation strength resulting from finite event numbers, which we assessed using surrogate correlation patterns from spatially shuffled events (see Methods). In nearly all cases at both P27–32 and P39–43, the strength of these long-range correlations was statistically significant relative to surrogate controls (23 of 25 vs shuffle at P27–32, 23 of 25 significant at P39–43 at *P* < 0.05), indicating that long range modular correlations continue to be a prominent feature of cortical networks in both sensory and non-sensory areas.

**Fig. 6:**
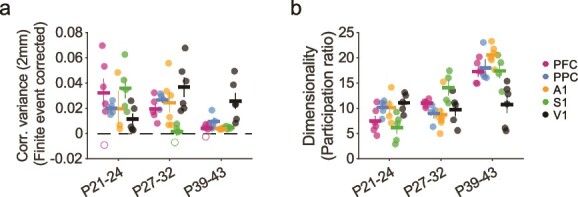
Maturation of correlation strength and dimensionality across cortical areas. a) Long-range correlations (estimated over distance interval 1.8 to 2.2 mm) remain statistically significant versus control across age in all areas, while showing a significant decline with age in all areas except V1. Closed circles indicate experiments with significant correlations relative to surrogate controls, whereas open circles are non-significant (*P* > 0.05). b) Dimensionality increases significantly with age in most cortical areas, except V1. Data from P21–24 animals in a to c replotted for comparison from (Powell et al. 2024). In all plots, circles show individual animals, horizontal lines and error bars show mean ± sem.

A critical advantage of this approach to quantifying a correlation strength is that it allows comparison across experiments at different ages and areas with different numbers of observed events. When comparing our data to that from animals at P21–24 published previously (Powell et al. 2024), we find that correlation variance at 2 mm changes significantly with age over all areas [Fig. 6a, ART, main effect of age: F(2) = 8.97, *P* = 4.03 × 10^−4^]. Although our data did not reveal a significant difference across areas, we did find a significant interaction of age and area, indicating differing developmental trajectories across regions [ART, main effect of area: F(4) = 1.17, *P* = 0.33; interaction age-area: F(8) = 4.01, *P* = 7.77 × 10^−4^]. Intriguingly, in S1, the largest decline occurred between P21–24 and P27–32 (post-hoc tests with Holm’s correction; t(58) = 4.21, *P* = 9.1 × 10^−3^, Supplemental Tables 9 and 10), whereas PFC, PPC and A1 showed larger declines in correlation strength by P39–43. In contrast to the other brain regions examined, correlation variance in V1 showed a trend toward increased strength with age (post-hoc tests with Holm’s correction; P21–24 vs P27–32 t(58) = −3.24, *P* = 0.16, Supplemental Table 10). Taken together, these results show that while both sensory and non-sensory areas exhibit long range correlations beyond the onset of sensory evoked responses, the developmental maturation of these correlated networks shows area-specific differences.

Spontaneous activity at early developmental time points (P21–24) is characterized by activity patterns that predominantly reside in a relatively small subspace of all possible activity patterns, which can be described with a relatively low number of linear dimensions (Powell et al. 2024). In order to determine whether the diversity of spontaneous activity patterns changed with age, we therefore computed the cross-validated dimensionality (Abbott et al. 2011; Stringer et al. 2019) of wide field spontaneous activity for each experiment, which provides a measure of the number of effective linear dimensions occupied by spontaneous activity patterns. We used this estimate of the diversity of activity patterns across events to examine how the complexity of cortical activity changes with age. Our results show that over all areas, the dimensionality of activity increases significantly with age [Fig. 6b, ART, main effect of age: F(2) = 49.33, *P* = 2.14 × 10^−13^] while remaining moderately low compared to the space of all possible activity patterns, consistent with the general decrease in correlation strength that we observe with age. Our results also reveal a significant interaction of age and area, indicating differing developmental trajectories across brain regions [ART, main effect of area: F(4) = 1.49, *P* = 0.22, interaction: F(8) = 6.98, *P* = 1.82 × 10^−6^]. Within individual areas, dimensionality increased significantly from P21–24 to P39–43 in PFC, A1, and S1 [post-hoc tests with Holm’s correction, PFC: t(60) = −5.61, *P* = 0.0001, A1: t(60) = −4.737, *P* = 0.0012, S1: t(60) = −5.821, *P* < 0.0001, Supplemental Tables 11 and 12), and increased from P27–32 to P39–49 in PPC (post-hoc tests with Holm’s correction, t(60) = −4.342, *P* = 0.0047]. In contrast, the dimensionality of activity in V1 appeared to change very little with age (post-hoc tests with Holm’s correction, P21–24 vs P39–43: t(60) = 0.043, *P* = 1.0). Collectively, these results show that millimeter-scale correlations and moderately low dimensional activity remain a central feature of spontaneous activity over development in both sensory and non-sensory cortices, and reflect an interplay between a common developmental trajectory and area-specific influences.

## Discussion

By examining multiple cortical areas serving diverse functions—ranging from primary sensory cortices to higher-order association areas—at different points in development, we found evidence for a common modular structure in spontaneous activity that remains a prominent organizing feature across areas. Our data build upon prior results which show that across multiple brain areas in early development before the transition to sensory-evoked activity, local circuits exhibit highly dense and modular activity that is coherent on both the local and millimeter-scale (Powell et al. 2024). The results presented here show that this common organization in the early cortex is followed by a developmental trajectory that reflects both common pathways of maturation across many diverse cortical areas as well as area-specific differences. In general, initially low dimensional and strongly correlated activity in both sensory and non-sensory cortices becomes increasingly rich and higher-dimensional with age, while retaining clear locally modular organization and long-range correlations over a period spanning major developmental milestones of eye-opening and ear canal opening. Notably, our results show that the central features of these networks—modular organization and millimeter-scale correlations—are conserved across cortical areas and ages, persisting well after this developmental transition to predominantly extrinsically driven activity.

However, the pattern of developmental changes was not universal across all cortical areas. In V1 correlation strength did not decline with age, and activity remained more low-dimensional than in other regions. The diversity of brain regions is generally thought to initially arise from broad genetically-specified gradients that are subsequently refined in an activity-dependent manner driven by area-specific feed-forward inputs (Sur and Rubenstein 2005; Cadwell et al. 2019). In this context, the relatively stronger modularity and millimeter-scale correlations seen in V1 compared to other cortical areas in older animals may reflect the emergence of a strong alignment of low-dimensional feed-forward input—encoding selectivity for edge orientation—with locally modular intracortical networks (Trägenap et al. 2023). Likewise, the continued strong long-range correlations in V1 may also be the contribution of the highly organized long-range horizontal projections that develop following eye-opening and specifically link co-tuned orientation columns (Gilbert and Wiesel 1989; Callaway and Katz 1990; Durack and Katz 1996; Bosking et al. 1997). Beyond these differences between V1 and other regions, our results show several additional intriguing differences between brain regions within a generally consistent pattern of developmental maturation. Across multiple metrics, activity in S1 shows large changes between P21–24 and P27–32, which appear to occur later (between P27–32 and P39–43) in other brain areas. These results suggest that the timing of maturation may differ across cortical areas, with S1 potentially progressing faster than other regions. Although additional experiments with fine scale sampling of developmental ages are needed to resolve this question, such an idea is consistent with results in mice showing the relatively earlier onset of sensory evoked and thalamocortically-driven responses in S1 (Antón-Bolaños et al. 2019) relative to V1 (Huberman et al. 2008).

Collectively, our findings provide new insights into the fundamental question of the developmental origins of diverse representations across cortical areas, suggesting that not only do highly diverse cortical areas start out with a highly similar millimeter-scale modular organization (Powell et al. 2024), but that this organization remains a prominent feature across areas even as the brain undergoes a developmental transition from internally-generated to externally-driven activity. These similarities in functional organization along with the increasing differences we observe across cortical areas over development suggest that functional diversity across brain areas may arise through the interaction of area-specific factors, potentially driven by the structure of area-specific feed-forward inputs (Sur and Rubenstein 2005; Cadwell et al. 2019), with this common modular organization.

Our experiments in this study were performed under light isoflurane anesthesia. This approach was selected both to provide consistency with the prior work with which we compare our results (Smith et al. 2018; Powell et al. 2024), but also to allow for imaging at early developmental time points which preclude the time required for habituation to head-fixation necessary for awake experiments. Our prior work in V1 did not identify any evidence for differences in the spatial structure of spontaneous activity between the awake and lightly anesthetized state (Smith et al. 2018). Likewise, varying the level of anesthesia did not alter cortex-wide functional connectivity between brain regions in the ferret (Fischer et al. 2018), however direct comparison with the awake state was not performed. Although these findings suggest that the spatial structure of activity that we observe in PFC, PPC, S1 and A1 are not the result of anesthesia, this remains to be tested directly outside V1. Notably, the same experiments in V1 which found no difference in the spatial structure of spontaneous activity between awake and anesthetized states found clear differences in the temporal structure of activity (Smith et al. 2018). Along these lines, the temporal patterns of activity we observe across brain regions and areas showing large spontaneous events are reminiscent of the Up/Down states observed during both sleep and anesthesia, which have been proposed to play a role in memory consolidation (Tukker et al. 2020). Thus, a direct examination of the spatial and temporal structure of spontaneous activity across ages and outside of V1 in both awake and sleep states, potentially leveraging recent advances in head mounted miniature microscopes (Hu et al. 2024), will be of great value in future work.

What circuit mechanisms might give rise to modular functional networks in multiple cortical areas? Prior work has suggested that in early development, when intracortical horizontal connections are thought to be primarily short-range (Durack and Katz 1996), recurrent local circuits can give rise to distributed and modular activity with long-range correlations through self-organizing mechanisms (Smith et al. 2018; Mulholland et al. 2024b). In such theoretical models (Ermentrout and Cowan 1979; Pinto and Ermentrout 2001), this modular structure arises through the interaction of local excitation and lateral inhibition (LE/LI), and predicts a tight coupling of activity in excitatory and inhibitory populations, which has been observed in vivo in V1 during early development (Mulholland et al. 2021). Such LE/LI mechanisms appear to be engaged in early V1 where unstructured optogenetic activation gives rise to modular cortical activity (Mulholland et al. 2024b). It is possible that a similar mechanism may operate in early development in other cortical regions, although this remains to be tested. Notably, in networks governed by LE/LI interactions, modular activity patterns are narrowly constrained to a characteristic spatial wavelength determined by these lateral interactions. In this context, our finding of highly similar spatial wavelengths across cortical areas (Fig. 4b) suggests that if such LE/LI mechanisms also operate in these areas, they likely involve lateral interactions with similar strengths and interaction distances, which show a similar pattern of changes with age. Such LE/LI models also suggest potential circuit-level mechanisms for the developmental changes we observe, as changing the relative extents or strengths of excitation and inhibition in these models can alter the amplitude and wavelength of the resulting modular activity patterns. Future studies directly measuring or manipulating the strength of these lateral interactions will be required to determine if developmental changes in LE/LI interactions shape the developmental maturation of modular networks. In addition, experiments applying direct optogenetic stimulation of the cortex (Mulholland et al. 2024a) across developmental periods could be used to further tease apart the relative contributions of input versus recurrent interactions in different brain regions, and determine if the area-specific changes we observe in both the structure and complexity of activity with age are possibly due to a developmental shift toward input-driven activity and the cortex becoming less strongly dominated by recurrent circuits.

In V1 of species with a columnar organization, long-range horizontal axons emerge over development and exhibit distributed and patchy arborizations that align spatially with functional modules representing stimulus features (Gilbert and Wiesel 1989; Callaway and Katz 1990; Bosking et al. 1997). Similar patchy horizontal axonal projections have been seen in a wide range of cortical areas in multiple species, and have been suggested to reflect a canonical wiring motif in the mature cortex (Douglas and Martin 2004; Muir and Douglas 2011). If such structured long-range connections emerge over development as a common feature across brain regions, they could serve to constrain and stabilize the large-scale correlated networks we observe in spontaneous activity in multiple cortical areas, thereby maintaining this millimeter-scale organization in the face of local sparsification and increased dimensionality. In addition, the strong long-range correlations in activity during development may serve to promote the developmental formation of such structured horizontal connections via Hebbian mechanisms. Finally, it is also possible that differences in the strength of such specific long-range horizontal connections between areas might also contribute to the relatively stronger millimeter-scale correlations seen in V1 at P39–43.

Our finding of a modular functional organization across many diverse cortical areas outside of early development is consistent with several prior observations. In mature V1 of primates and carnivores, the presence of a modular functional organization is well documented in the structure of visually-evoked feature maps (Hubel and Wiesel 1968; Blasdel and Salama 1986; Weliky et al. 1996; Issa et al. 2000; Kara and Boyd 2009; Smith et al. 2015a). This modular organization is also present in spontaneous activity (Kenet et al. 2003; O’Hashi et al. 2018; Smith et al. 2018), which we also see in our results from V1. Likewise, our finding that such distributed modular correlated networks are present in other sensory areas (A1, S1) well after the transition to extrinsically-driven activity is consistent with the presence of modular organization of feature representations reported in these areas (Sur et al. 1981; Recanzone et al. 1999; Schreiner et al. 2000; Friedman et al. 2004; Atencio and Schreiner 2012). Furthermore, in the mature primate PFC, anatomical projections corresponding to distinct information streams show spatial clustering (Goldman-Rakic and Schwartz 1982; Kritzer and Goldman-Rakic 1995). Whether the correlated networks we observe in spontaneous activity in these areas correspond to such representations—as they do in V1 (Kenet et al. 2003; O’Hashi et al. 2018; Smith et al. 2018)—remains to be determined. Neural activity in PFC has been linked to executive control and working memory (Fuster 2000), raising the intriguing possibility that different modular patterns could represent distinct rule sets of action contingencies. Addressing this would require implementing cognitive control behavioral tasks in conjunction with head-mounted wide field miniscopes, which could potentially reveal the association of modular activity patterns in PFC to specific behavioral events. In addition, it is also possible that the presence of modular networks with similar dimensionality and spatial scales across brain regions in early development serves to coordinate activity across regions into the larger-scale multi-area functional networks as observed in brain-wide fMRI in humans (Yeo et al. 2011).

The question of whether functional modules such as those we observe in correlated spontaneous activity play a role in cortical computations has long been the subject of investigation. Modular organization can lead to efficient networks by minimizing connection distances between functionally coupled neurons (Koulakov and Chklovskii 2001), and can also lead to computational advantages (Meunier et al. 2010). Our finding that modular networks in most brain regions weaken and become higher dimensional with age suggests that such an organization may play a developmental role in coarsely grouping neurons into functional units in early development before giving way to more locally-diverse organization. Whether the modules we observe in different cortical areas in animals well past eye-opening reflect a persistent feature of developmental network organization, or rather contribute directly to cortical computation, remains to be determined and could potentially vary across different cortical areas.

## Supplementary Material

Powell_2024_Dev_Revision_Sup_Mat_bhaf007

Powell_2024_Dev_Revision_Sup_Mat_Tables_bhaf007
