# Supplementary material for: Developmental maturation of millimeter-scale functional networks across brain areas
Source: Cereb Cortex. 2025 Jan 25;35(2):bhaf007. doi: 10.1093/cercor/bhaf007 (PMC11795307; doi:10.1093/cercor/bhaf007)
Supplement: Powell_2024_Dev_Revision_Sup_Mat_bhaf007 [file powell_2024_dev_revision_sup_mat_bhaf007.pdf]

Supplementary Materials

Developmental maturation of millimeter-scale functional networks across brain areas

Abbreviated title: Maturation of millimeter-scale networks

Nathaniel J. Powell[1*], Bettina Hein[2*], Deyue Kong[3,4,5], Jonas Elpelt[3,4], Haleigh N. Mulholland[1], Ryan A. Holland[1], Matthias Kaschube[3,4+], Gordon B. Smith[1+]
1. Optical Imaging and Brain Sciences Medical Discovery Team, Department of Neuroscience, University of Minnesota, Minneapolis, MN, USA
2. Center for Theoretical Neuroscience, Columbia University, New York, NY, USA
3. Frankfurt Institute for Advanced Studies, Frankfurt am Main, Germany
4. Goethe University Frankfurt, Department of Computer Science and Mathematics, Frankfurt am Main, Germany
5. International Max Planck Research School for Neural Circuits, Frankfurt, Germany
*Contributed equally.
[+]Co-supervised work.

Corresponding author: Gordon Smith
Email: gbsmith@umn.edu
Address:
1-311C CMRR
2021 6th Street S.E.
Minneapolis, MN 55455, USA

Supplemental Figures

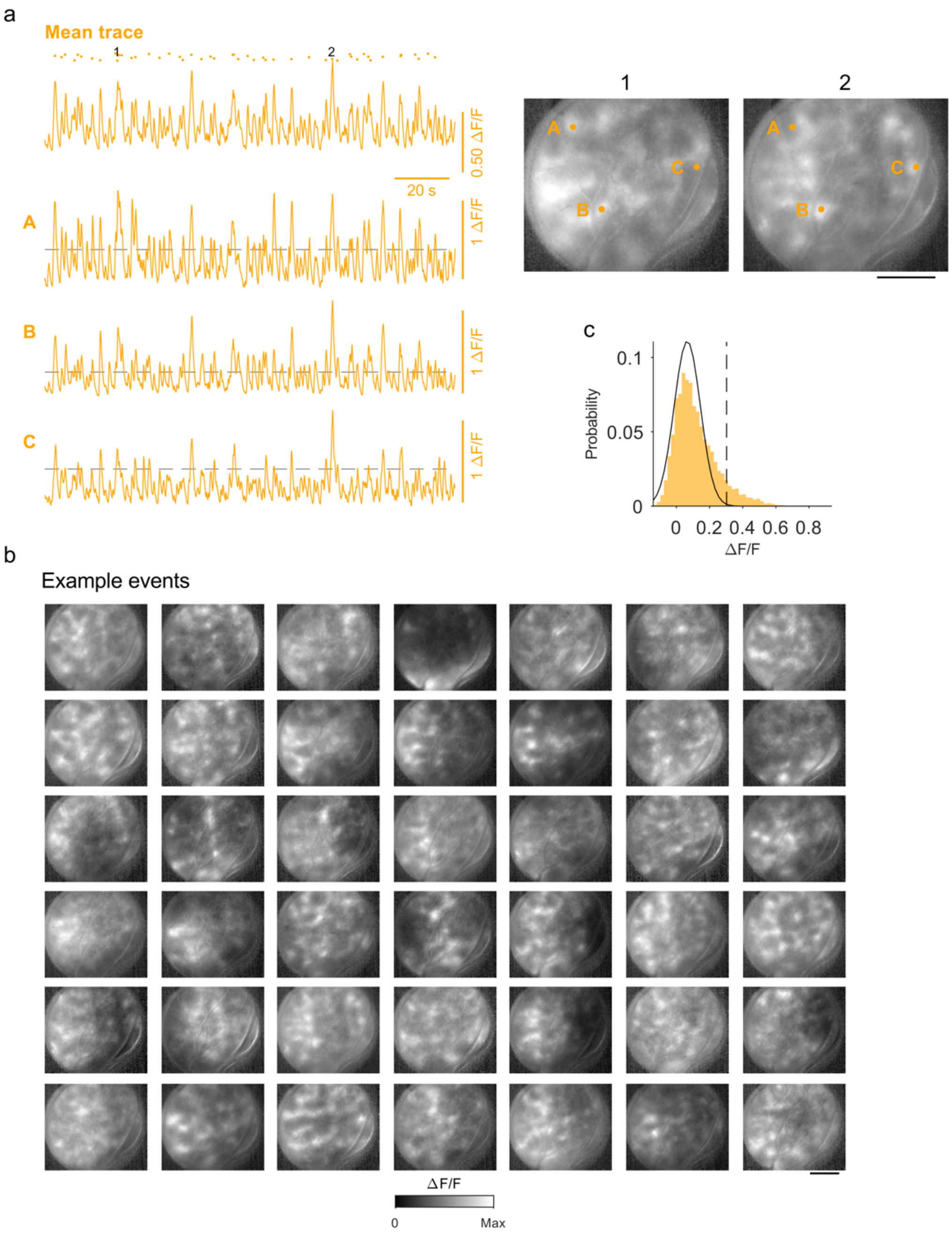

Supplemental Figure 1: Individual pixel traces and event detection in A1 at P32. a. (*Right*) Mean trace over all pixels in ROI (top) and traces from 3 individual pixels at locations shown in images to *Left*. Data from area A1 at P32, same experiment as shown Figure 2a. Numbers indicate event images shown to left. Dots above mean trace indicate identified events. Dashed line indicates active pixel threshold for each pixel trace shown. **b**. Example events from time period shown in (a). Scale bars for images in (a) and (b): 1 mm. **c**. Distribution of ΔF/F values for pixel in A in (a). Black line is standard gaussian fit to the data. Black dashed line indicated threshold at 3 standard deviations above the mean of this gaussian fit.

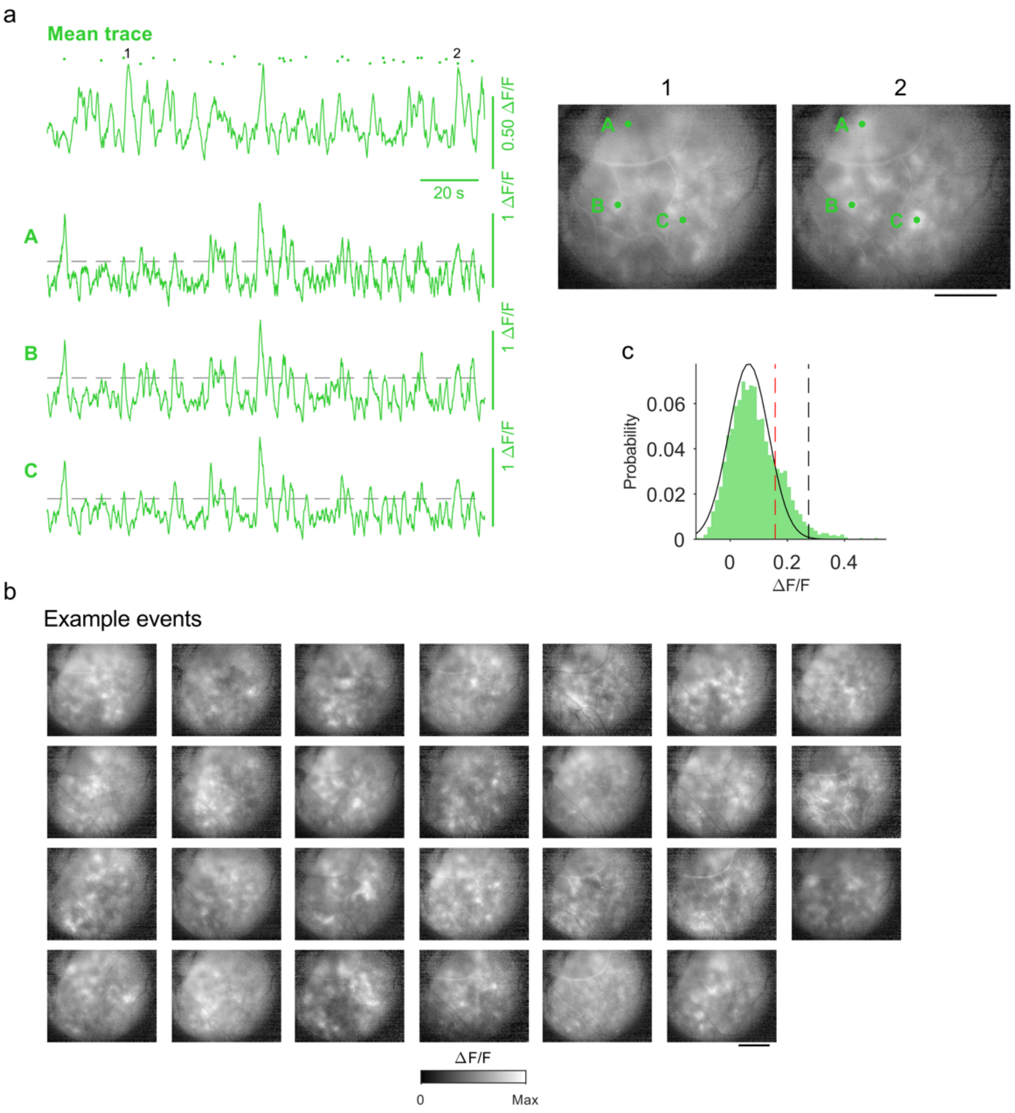

Supplemental Figure 2: Individual pixel traces and event detection in S1 at P39. a. (*Right*) Mean trace over all pixels in ROI (top) and traces from 3 individual pixels at locations shown in images to *Left*. Data from area S1 at P39, same experiment as shown Figure 3a. Numbers indicate event images shown to left. Dots above mean trace indicate identified events. Dashed line indicates active pixel threshold for each pixel trace shown. **b**. Example events from time period shown in (a). Scale bars for images in (a) and (b): 1 mm. **c**. Distribution of ΔF/F values for pixel in A in (a). Black line is standard gaussian fit to the data. Black dashed line indicated threshold at 3 standard deviations above the mean of this gaussian fit. Red dashed line indicates 80th percentile threshold.

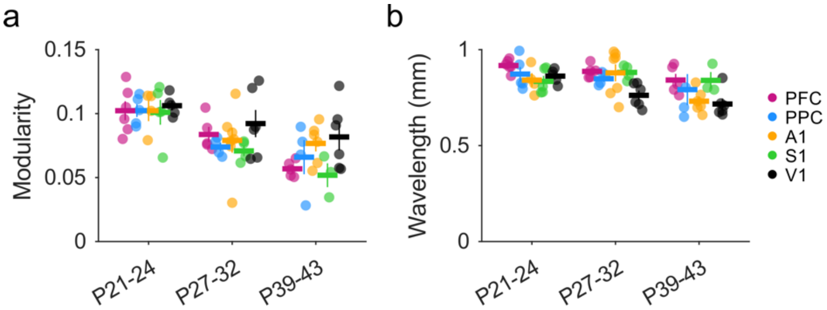

Supplemental figure 3: Choice of pixel-wise event detection threshold does not affect measurement of developmental maturation of modular spontaneous activity across cortical areas. a-b. Modularity (a) and wavelength (b) show similar changes with development when a pixel-wise activity threshold of 3 standard deviations is used (see Methods; compare to Figure 4a,b). In all plots, circles show individual animals, horizontal lines and error bars show mean ± sem.
