# Supplementary material for: Developmental maturation of millimeter-scale functional networks across brain areas
Source: Cereb Cortex. 2025 Jan 25;35(2):bhaf007. doi: 10.1093/cercor/bhaf007 (PMC11795307; doi:10.1093/cercor/bhaf007)
Supplement: Powell_2024_Dev_Revision_Sup_Mat_Tables_bhaf007 [file powell_2024_dev_revision_sup_mat_tables_bhaf007.pdf]

Supplementary Tables

Developmental maturation of millimeter-scale functional networks across brain areas

Abbreviated title: Maturation of millimeter-scale networks

Nathaniel J. Powell[1*], Bettina Hein[2*], Deyue Kong[3,4,5], Jonas Elpelt[3,4], Haleigh N. Mulholland[1], Ryan A. Holland[1], Matthias Kaschube[3,4+], Gordon B. Smith[1+]
1. Optical Imaging and Brain Sciences Medical Discovery Team, Department of Neuroscience, University of Minnesota, Minneapolis, MN, USA
2. Center for Theoretical Neuroscience, Columbia University, New York, NY, USA
3. Frankfurt Institute for Advanced Studies, Frankfurt am Main, Germany
4. Goethe University Frankfurt, Department of Computer Science and Mathematics, Frankfurt am Main, Germany
5. International Max Planck Research School for Neural Circuits, Frankfurt, Germany
*Contributed equally.
[+]Co-supervised work.

Corresponding author: Gordon Smith
Email: gbsmith@umn.edu
Address:
2021 6th Street S.E.
Minneapolis, MN 55455, USA

Supplemental Table 1: Event modularity mean (sem)

Area	P21 - 24	P27 - 32	P39 - 43
PFC	0.102 (0.007), n=6	0.084 (0.006), n=5	0.055 (0.001), n=5
PPC	0.102 (0.005), n=5	0.074 (0.004), n=4	0.072 (0.011), n=4
A1	0.102 (0.008), n=4	0.079 (0.010), n=7	0.076 (0.004), n=6
S1	0.101 (0.010), n=5	0.071 (0.004), n=4	0.052 (0.005), n=4
V1	0.106 (0.004), n=5	0.092 (0.011), n=6	0.083 (0.008), n=6

Supplemental Table 2: Event modularity: pairwise p-values (Holm's correction)

		P21-24					P27-31					P39-43				
		PFC	PPC	A1	S1	V1	PFC	PPC	A1	S1	V1	PFC	PPC	A1	S1	V1
P21-24	PFC															
	PPC	1.000														
	A1	1.000	1.000													
	S1	1.000	1.000	1.000												
	V1	1.000	1.000	1.000	1.000											
P27-31	PFC	1.000	1.000	1.000	1.000	1.000										
	PPC	0.505	0.493	0.996	0.957	0.239	1.000									
	A1	1.000	1.000	1.000	1.000	0.639	1.000	1.000								
	S1	0.184	0.183	0.431	0.394	0.076	1.000	1.000	1.000							
	V1	1.000	1.000	1.000	1.000	1.000	1.000	1.000	1.000	1.000						
P39-43	PFC	**0.001**	**0.001**	**0.003**	**0.002**	**0.000**	0.504	1.000	0.353	1.000	**0.025**					
	PPC	0.639	0.608	1.000	1.000	0.305	1.000	1.000	1.000	1.000	1.000	1.000				
	A1	0.349	0.349	0.797	0.719	0.143	1.000	1.000	1.000	1.000	1.000	1.000	1.000			
	S1	**0.001**	**0.001**	**0.006**	**0.004**	**0.001**	0.639	1.000	0.493	1.000	**0.044**	1.000	1.000	1.000		
	V1	1.000	1.000	1.000	1.000	1.000	1.000	1.000	1.000	1.000	1.000	0.290	1.000	1.000	0.394	

Supplemental Table 3: Event wavelength mean (sem)

Area	P21 - 24	P27 - 32	P39 - 43
PFC	0.917 (0.013), n=6	0.887 (0.015), n=5	0.876 (0.025), n=5
PPC	0.873 (0.037), n=5	0.848 (0.017), n=4	0.780 (0.078), n=4
A1	0.841 (0.036), n=4	0.879 (0.045), n=7	0.722 (0.018), n=6
S1	0.835 (0.029), n=5	0.882 (0.019), n=4	0.850 (0.032), n=4
V1	0.862 (0.016), n=5	0.762 (0.025), n=6	0.701 (0.033), n=6

Supplemental Table 4: Event wavelength: Cross area pairwise p-values (Holm's correction)

	PFC	PPC	A1	S1	V1
PFC					
PPC	0.065				
A1	**0.014**	0.713			
S1	0.369	0.713	0.484		
V1	**0.000**	0.140	0.248	**0.014**	

Supplemental table 5: Event wavelength: pairwise p-values (Holm's correction)

		P21-24					P27-31					P39-43				
		**PFC**	**PPC**	**A1**	**S1**	**V1**	**PFC**	**PPC**	**A1**	**S1**	**V1**	**PFC**	**PPC**	**A1**	**S1**	**V1**
**P21-24**	**PFC**															
	**PPC**	1.000														
	**A1**	1.000	1.000													
	**S1**	1.000	1.000	1.000												
	**V1**	1.000	1.000	1.000	1.000											
**P27-31**	**PFC**	1.000	1.000	1.000	1.000	1.000										
	**PPC**	1.000	1.000	1.000	1.000	1.000	1.000									
	**A1**	1.000	1.000	1.000	1.000	1.000	1.000	1.000								
	**S1**	1.000	1.000	1.000	1.000	1.000	1.000	1.000	1.000							
	**V1**	**0.021**	1.000	1.000	1.000	1.000	0.422	1.000	0.575	0.924						
**P39-43**	**PFC**	1.000	1.000	1.000	1.000	1.000	1.000	1.000	1.000	1.000	1.000					
	**PPC**	0.464	1.000	1.000	1.000	1.000	1.000	1.000	1.000	1.000	1.000	1.000				
	**A1**	**0.001**	0.260	1.000	1.000	0.352	**0.045**	1.000	0.052	0.129	1.000	0.129	1.000			
	**S1**	1.000	1.000	1.000	1.000	1.000	1.000	1.000	1.000	1.000	1.000	1.000	1.000	1.000		
	**V1**	**0.001**	0.200	1.000	1.000	0.272	**0.034**	1.000	**0.039**	0.100	1.000	0.100	1.000	1.000	1.000	

Supplemental Table 6: Module amplitude mean (sem)

Area	P21 - 24	P27 - 32	P39 - 43
PFC	2.72 (0.19), n=6	2.15 (0.14), n=5	1.77 (0.04), n=6
PPC	2.92 (0.10), n=5	2.03 (0.10), n=6	1.81 (0.09), n=6
A1	3.09 (0.16), n=4	1.94 (0.12), n=7	1.68 (0.05), n=6
S1	2.38 (0.13), n=5	1.64 (0.09), n=5	1.40 (0.03), n=4
V1	3.74 (0.28), n=5	2.20 (0.11), n=3	1.94 (0.04), n=5

Supplemental table 7: Module amplitude: Cross area pairwise p-values (Holm's correction)

	PFC	PPC	A1	S1	V1
**PFC**					
**PPC**	0.948				
**A1**	0.883	0.883			
**S1**	**0.001**	**0.001**	**0.015**		
**V1**	**0.003**	**0.003**	**0.000**	**0.000**	

Supplemental table 8: Module amplitude: pairwise p-values (Holm's correction)

		P21-24					P27-31					P39-43				
		PFC	PPC	A1	S1	V1	PFC	PPC	A1	S1	V1	PFC	PPC	A1	S1	V1
P21-24	PFC															
	PPC	1.000														
	A1	1.000	1.000													
	S1	1.000	1.000	0.901												
	V1	1.000	1.000	1.000	0.104											
P27-31	PFC	0.252	**0.021**	**0.018**	1.000	**0.001**										
	PPC	**0.032**	**0.002**	**0.002**	0.901	**0.000**	1.000									
	A1	**0.001**	**0.000**	**0.000**	0.090	**0.000**	1.000	1.000								
	S1	**0.000**	**0.000**	**0.000**	**0.000**	**0.000**	**0.010**	**0.035**	0.285							
	V1	1.000	0.390	0.292	1.000	**0.035**	1.000	1.000	1.000	**0.009**						
P39-43	PFC	**0.000**	**0.000**	**0.000**	**0.002**	**0.000**	0.252	0.720	1.000	1.000	0.153					
	PPC	**0.000**	**0.000**	**0.000**	**0.004**	**0.000**	0.491	1.000	1.000	1.000	0.285	1.000				
	A1	**0.000**	**0.000**	**0.000**	**0.000**	**0.000**	**0.015**	0.054	0.445	1.000	**0.013**	1.000	1.000			
	S1	**0.000**	**0.000**	**0.000**	**0.000**	**0.000**	**0.000**	**0.000**	**0.003**	1.000	**0.000**	0.199	0.092	1.000		
	V1	**0.016**	**0.001**	**0.001**	0.491	**0.000**	1.000	1.000	1.000	0.155	1.000	1.000	1.000	0.246	**0.002**	

Supplemental Table 9: Correlation strength - 2mm mean (sem)

Area	P21 - 24	P27 - 32	P39 - 43
PFC	0.031 (0.012), n=6	0.019 (0.005), n=5	0.003 (0.002), n=5
PPC	0.014 (0.003), n=5	0.026 (0.004), n=4	0.015 (0.006), n=4
A1	0.022 (0.018), n=3	0.027 (0.007), n=6	0.004 (0.000), n=5
S1	0.033 (0.005), n=5	0.002 (0.003), n=4	0.005 (0.001), n=4
V1	0.011 (0.006), n=5	0.036 (0.007), n=6	0.023 (0.007), n=6

Supplemental table 10: Correlation strength - 2mm: pairwise p-values (Holm's correction)

		P21-24					P27-31					P39-43				
		PFC	PPC	A1	S1	V1	PFC	PPC	A1	S1	V1	PFC	PPC	A1	S1	V1
P21-24	PFC															
	PPC	1.000														
	A1	1.000	1.000													
	S1	1.000	1.000	1.000												
	V1	1.000	1.000	1.000	0.259											
P27-31	PFC	1.000	1.000	1.000	1.000	1.000										
	PPC	1.000	1.000	1.000	1.000	0.899	1.000									
	A1	1.000	1.000	1.000	1.000	1.000	1.000	1.000								
	S1	**0.050**	0.346	1.000	**0.009**	1.000	0.422	**0.047**	0.162							
	V1	1.000	1.000	1.000	1.000	0.165	1.000	1.000	1.000	**0.005**						
P39-43	PFC	0.102	0.710	1.000	**0.018**	1.000	0.865	0.097	0.328	1.000	**0.009**					
	PPC	1.000	1.000	1.000	0.598	1.000	1.000	1.000	1.000	1.000	0.422	1.000				
	A1	**0.026**	0.234	1.000	**0.004**	1.000	0.287	**0.028**	0.097	1.000	**0.002**	1.000	1.000			
	S1	0.164	0.920	1.000	**0.032**	1.000	1.000	0.142	0.467	1.000	**0.019**	1.000	1.000	1.000		
	V1	1.000	1.000	1.000	1.000	1.000	1.000	1.000	1.000	0.060	1.000	0.124	1.000	**0.032**	0.194	

Supplemental Table 11: Dimensionality mean (sem)

Area	P21 - 24	P27 - 32	P39 - 43
PFC	7.48 (1.01), n=6	11.01 (0.32), n=5	17.23 (1.04), n=5
PPC	10.17 (0.66), n=5	8.95 (0.92), n=4	17.96 (1.68), n=4
A1	10.13 (1.54), n=4	8.76 (1.19), n=7	20.47 (0.79), n=6
S1	6.16 (1.21), n=5	14.03 (1.58), n=4	17.38 (1.57), n=4
V1	11.07 (0.83), n=5	9.72 (1.37), n=5	10.74 (1.66), n=6

Supplemental Table 12: Dimensionality: pairwise p-values (Holm's correction)

		P21-24					P27-31					P39-43				
		**PFC**	**PPC**	**A1**	**S1**	**V1**	**PFC**	**PPC**	**A1**	**S1**	**V1**	**PFC**	**PPC**	**A1**	**S1**	**V1**
**P21-24**	**PFC**															
	**PPC**	1.000														
	**A1**	1.000	1.000													
	**S1**	1.000	0.718	1.000												
	**V1**	0.668	1.000	1.000	0.152											
**P27-31**	**PFC**	0.596	1.000	1.000	0.134	1.000										
	**PPC**	1.000	1.000	1.000	1.000	1.000	1.000									
	**A1**	1.000	1.000	1.000	1.000	1.000	1.000	1.000								
	**S1**	**0.013**	1.000	1.000	**0.002**	1.000	1.000	0.239	0.060							
	**V1**	1.000	1.000	1.000	1.000	1.000	1.000	1.000	1.000	0.758						
**P39-43**	**PFC**	**0.000**	0.065	0.054	**0.000**	0.345	0.387	**0.004**	**0.000**	1.000	**0.018**					
	**PPC**	**0.000**	0.063	0.052	**0.000**	0.314	0.345	**0.005**	**0.001**	1.000	**0.018**	1.000				
	**A1**	**0.000**	**0.001**	**0.001**	**0.000**	**0.011**	**0.013**	**0.000**	**0.000**	1.000	**0.000**	1.000	1.000			
	**S1**	**0.000**	0.084	0.068	**0.000**	0.396	0.441	**0.007**	**0.001**	1.000	**0.026**	1.000	1.000	1.000		
	**V1**	1.000	1.000	1.000	0.333	1.000	1.000	1.000	1.000	1.000	1.000	0.077	0.075	**0.001**	0.103	
